# Salinity indicators in sediment through the fluvial-to-marine transition (Fraser River, Canada)

**DOI:** 10.1038/s41598-022-18466-4

**Published:** 2022-08-22

**Authors:** Shahin E. Dashtgard, Aihua Wang, Vera Pospelova, Pei-Ling Wang, Andrew La Croix, Korhan Ayranci

**Affiliations:** 1 Earth Sciences, Simon Fraser University, Burnaby, BC V5A 1S6 Canada; 2 Nanjing Center, China Geological Survey, Nanjing, 210016 China; 3 Earth and Environmental Sciences, University of Minnesota, Minneapolis, MN 55455 USA; 4 Institute of Oceanography, National Taiwan University, Taipei, Taiwan; 5 Earth and Environmental Sciences, School of Science, University of Waikato, Hamilton, 3240 New Zealand; 6 Petroleum Engineering and Geosciences, King Fahd University of Petroleum and Minerals, Dhahran, 31261 Saudi Arabia

**Keywords:** Sedimentology, Geochemistry

## Abstract

Many sediment attributes have been proposed as proxies for determining salinity conditions under which sediment is deposited, and six attributes (Sr/Ba-HAc, Sr/Ba-NH_4_Ac, δ^13^C_org_, C/N, and the relative abundances and concentrations of dinoflagellate cysts) are compared here. In this paper, sediment attributes from the Fraser River Delta, Canada and surrounding coastal areas are compared by depositional position along the fluvial-to-marine transition, by salinity, and by sedimentological characteristics. Along the fluvial-to-marine transition, most attributes exhibit distinct trends between parts of the river that experience *sustained* marine water (saltwater) influence over seasonal and tidal timeframes, and parts that experience only freshwater or periodic saltwater influence. No attributes are reliable indicators of depositional position where saltwater incursion is short lived or where water is fresh. Where marine influence is sustained, Sr/Ba-HAc and Sr/Ba-NH_4_Ac are the most reliable positional indicators along the fluvial-to-marine transition. When compared strictly to salinity, Sr/Ba-HAc, Sr/Ba-NH_4_Ac, and δ^13^C_org_ all correlate predictably except in delta front and prodelta settings. Our data show that all six sediment attributes are heavily impacted by river-derived sedimentation, and it is not appropriate to compare values from strongly river-influenced settings (e.g., deltas) with those from weakly river-influenced settings (e.g., bays and estuaries).

## Introduction

The salinity of water under which sediment is deposited significantly influences the character of the sediment deposited therein^1–4^. For example, mud beds generally extend laterally over longer distances in brackish, shallow-water settings (e.g., estuaries and deltas) than in adjacent freshwater and saltwater settings^1,5^ suggesting that paleosalinity can be used to constrain mudstone-bed lengths in the sedimentary record. This, in turn, directly impacts the recovery of fluids from those rocks and sediments.

A wide range of sediment attributes, including both biological and geochemical attributes have been proposed as proxies for estimating paleosalinity in sediments and sedimentary rocks, with data used to develop and support these relations derived mainly from studies of modern depositional systems. Biological proxies include, but are not limited to, ichnology^6–8^, calcareous microfossils^9^, and organic-walled dinoflagellate cyst abundances^10–13^ and morphologies^14^. Geochemical proxies include, but are not limited to, δ^13^C_org_ and/or C/N^10,15^, B/Ga^16,17^, and Sr/Ba^18,19^. Quantitative comparisons of biological and geochemical proxies in estuaries and deltas are rare^16^ so the accuracy and utility of different proxies under differing depositional and preservational conditions is poorly constrained. In this study, we compare six sediment attributes, including Sr/Ba (derived using two different extraction methods), δ^13^C_org_, C/N, and the concentrations and relative abundances of dinoflagellate cysts (referred to herein as dinocysts) to (1) depositional position along the fluvial-to-marine transition (FMT) of the Fraser River and Delta, Canada, (2) salinity, and (3) physical sedimentological characteristics (i.e., mean grain size, mud content, and clay content). The FMT is defined herein as extending from the seaward limit of the prodelta to the landward limit of the tidal backwater in the Fraser River. We then comment on the utility of each sediment attribute as a proxy for estimating salinity and predicting depositional position along the FMT, and the potential application of these attributes for estimating paleosalinity in the sedimentary record.

Sr/Ba is used as a salinity indicator because terrestrial sediment is typically enriched in Ba and poor in Sr when compared to marine sediment and vice versa^19^. In theory, Sr/Ba values < 1.0 are typical of terrestrial sediment and values > 1.0 are typical of marine sediment^19,20^; however, the concentration of Sr in most terrigenous clastic sediments (or rocks) is 100–300 mg kg^−1^ and the concentration of Ba is 300–750 mg kg^−1^ such that Sr/Ba in both marine and terrestrial *terrigenous*, bulk clastic sediments (rocks) is typically < 1.0^16,19,21^. Recently, Wang, et al.^19^ argued against using bulk-rock/sediment Sr/Ba for discriminating between marine and terrestrial sedimentary environments. Instead, they proposed two new Sr/Ba ratios derived through selective extraction of Ba and Sr with both 10% acetic acid (Sr/Ba-HAc) and 1 M of ammonium acetate (Sr/Ba-NH_4_Ac). These selective extraction techniques yielded a strong linear correlation between Sr/Ba-HAc and Sr/Ba-NH_4_Ac and salinity in laboratory-controlled experiments, and a reasonable correlation with salinity in the Yangtze River Delta, China.

Stable carbon isotopes (δ^13^C_org_) and C/N of organic carbon have also been used to establish salinity gradients because of differences in δ^13^C_org_ and C/N of terrestrial organic carbon sources and their marine counterparts^10,15,22–24^. Within the channelized extent of the FMT, mixing of terrestrial- and marine-sourced carbon occurs as a result of the backwater effect, reversed current flow during flood tides, and potentially due to flow separation in the freshwater tidal reach^10^.

Palynological indicators are also commonly employed to resolve salinity and rely partly on the same mechanisms that produce δ^13^C_org_ trends. Key palynological indicators of salinity conditions include the concentration of dinocysts and their abundance relative to pollen and spores^10,11,24,25^.

**Figure 1:**
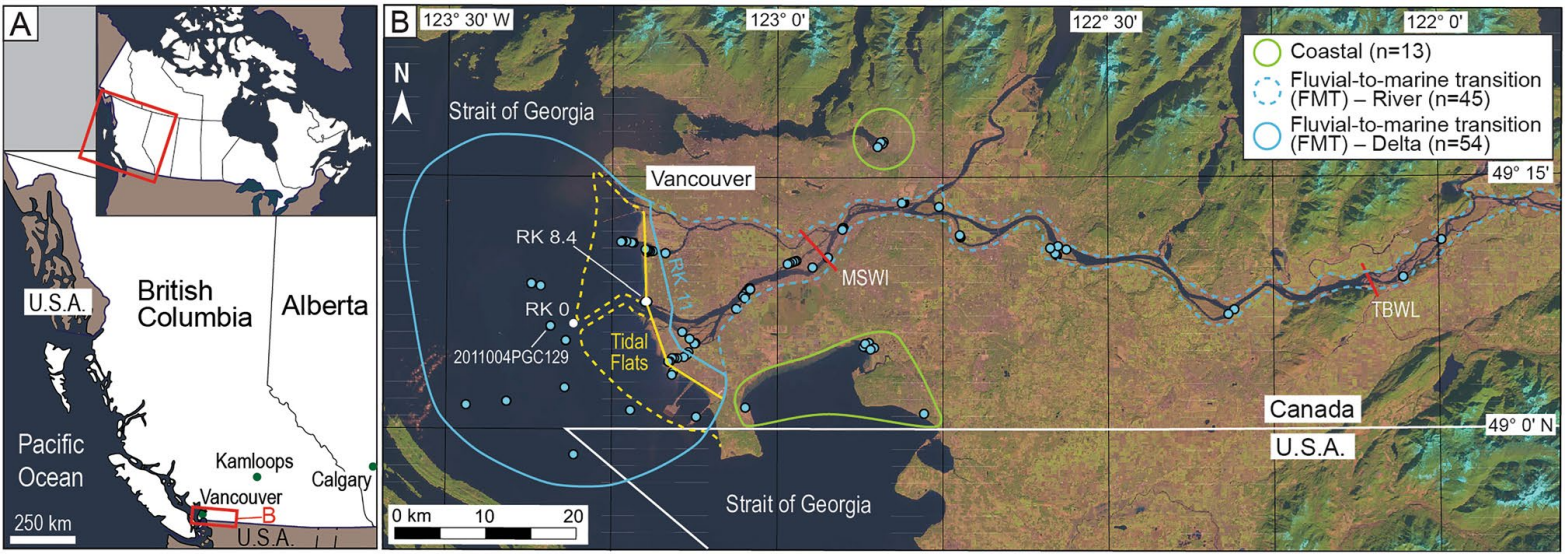
(**A**) Position of the Fraser River Delta in Canada and British Columbia. (**B**) Blue dots mark the 111 unique sample positions. The blue polygon encompasses samples from the fluvial-to-marine transition and include samples from the River (dashed blue line) and Delta (solid blue line). The division between River and Delta samples is at river km (RK) 11 (see "Discussion"). The green polygons encompass samples from Coastal areas, and the dashed yellow lines marks the approximate seaward limit of the tidal flats. The solid yellow line marks the approximate position of the dikes along the margin of the Fraser Delta and this line is used as a baseline for assigning river km values to samples across the tidal flats and in the Strait of Georgia (i.e., Delta). The position of Sand Heads (RK 0), the dyke upstream of Sand Heads (RK 8.4), maximum saltwater incursion (MSWI; ~ RK 30)^37–40^, and tidal backwater limit (TBWL; RK 102) are shown (image Source: Landsat 8; USGS and NASA).

In this study, samples were acquired along the FMT of the Fraser River, Canada and from surrounding coastal areas in the Strait of Georgia (Fig. 1). The Fraser River is a high-gradient system that transports an average 17 × 10^9^ kg yr^−1^ of sediment to the Strait of Georgia, of which ~ 36% is sand^26,27^. Tides are mesotidal and river discharge ranges from 1 000 to 15 200 m^3^ s^−1^ (mean: 2 710 m^3^ s^−1^). Tidal incursion up the Fraser River is approximately 30 km under low flow conditions (< 2 000 m^3^ s^−1^; Fig. 1B) and is pushed completely out of the river under high flow conditions (freshet; > 8 000 m^3^ s^−1^). The tidal backwater limit (limit of tidal modulation of river flow) is situated at approximately 102 km inland^28,29^.

## Results

**Figure 2:**
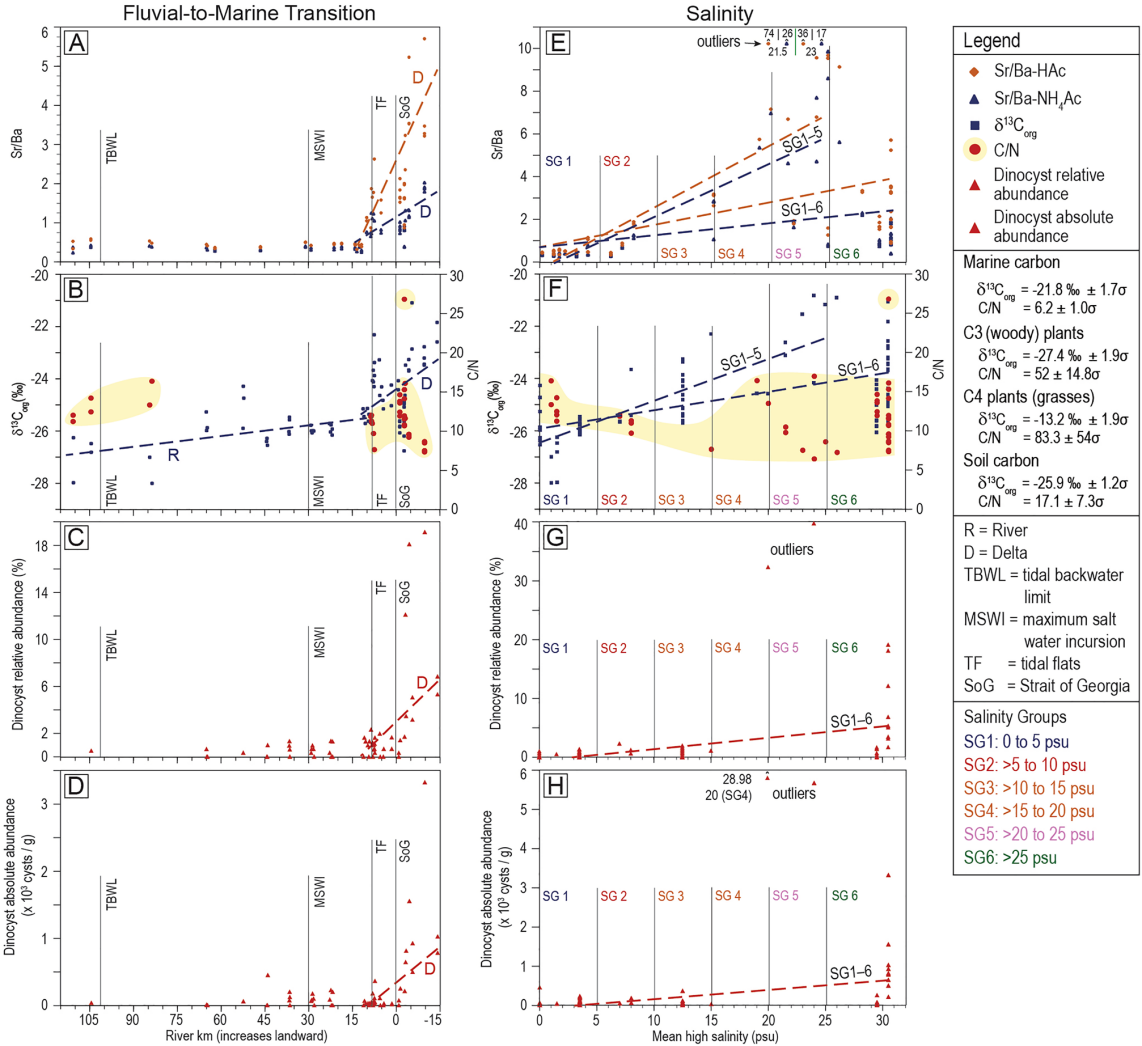
Graphs of Sr/Ba, δ^13^C_org_, C/N, and dinocyst relative abundances and concentrations along the FMT (**A–D**) and as a function of salinity (**E–H**). Data are available in Supplementary Data File A. δ^13^C_org_ and C/N values for different sources of organic matter in the legend are derived from Dashtgard, et al.^22^. The linear regression equations and R^2^ values are shown in Table 1 (excluding equations for “shallow” samples). The two outliers in (**E**) are Coastal samples that are near each other and represent the same depositional environment (MB-21-S3 and X-U1-1, Supplementary Data File A). The two outliers in (**G**) and (**H**) are also Coastal samples but are geographically distinct (MB-21S2 and PoM21-S2, Supplementary Data File A). These outliers are included to illustrate the distribution of data but are excluded in linear regression calculations (see "Methods").

This study includes both new and published data comprising 111 unique samples and 14 repeat analyses (n = 125; Supplementary Data File A). Of the 111 samples, 98 are from the FMT of Fraser River and an additional 13 are from nearby coastal areas (Fig. 1B). The mean high salinity value for each sample and its depositional position relative to Sand Heads (49.102901° N, 123.300347° W; Fig. 1B) are determined. Depositional position is the site of sediment deposition along the FMT of the Fraser River and is measured in river km^30^; positive values indicate a sample was collected upstream of Sand Heads (i.e., within channels or on the tidal flats). The positions of samples from the tidal flats and Strait of Georgia seafloor are measured perpendicular to the dike (relative to RK 8.4, Fig. 1B). For samples from the Coastal, FMT–River, and tidal flats in the FMT–Delta regions (Fig. 1), mean high salinity is derived from surface water, and is either 1) the average of measured salinity values taken at maximum tidal incursion and during low river flow, or 2) an estimate based on previous studies^31–38^ and/or measurements taken at different stages of tidal cycles or river flow. Consequently, mean high salinity values recorded for samples are representative and not absolute. Mean high salinity values for FMT–Delta samples from the Strait of Georgia seafloor are means derived from long-term records taken close to the seafloor^39^. Samples are grouped into Salinity Groups (SG #) based on the mean high salinity under which those sediments were deposited (Fig. 2). Mean grain size, and the sand (62.5–2 000 μm), silt (3.91–62.5 μm), clay (< 3.91 μm), and mud (< 62.5 μm) content of samples is also measured and used as a basis for comparison (Supplementary Data File A).

### Depositional position indicators in the fluvial-to-marine transition

Qualitative interpretations of graphs reveal that all six sediment attributes show some relation to depositional position within the FMT (Fig. 2A–D). In nearly all cases there is a significant break in values between samples in the river (RK 110.6 to RK > 11; referred to herein as River; Fig. 1B) and those derived from various parts of the lower river and delta (RK ≤ 11 to RK − 14.2; referred to herein as Delta). Trends and analyses are presently separately for River and Delta samples in the FMT. As well, in the Delta, distinctions are made between “shallow” samples (from the upper 1 m of the sediment pile) and ones taken at depth in cores.

Sr/Ba-HAc is 0.45 ± 0.05 (mean ± standard deviation; n = 20) and Sr/Ba-NH_4_Ac is 0.35 ± 0.05 (n = 20; Fig. 2A) through the River region of the FMT. Both ratios increase linearly through the Delta region of the FMT, and Sr/Ba-HAc (y = − 0.125x + 2.14, R^2^ = 0.52) increases at a rate 3 times greater than Sr/Ba-NH_4_Ac (y = − 0.042x + 1.0, R^2^ = 0.45; Table 1). If only surface and near surface samples are included, the correlation improves between depositional position and Sr/Ba-HAc (R^2^ = 0.75) and Sr/Ba-NH_4_Ac (R^2^ = 0.58).

**Table 1:** Summary of statistics between the six sediment attributes and both depositional position in the FMT and mean high salinity.

Comparison of trends by river km (RK)-fluvial-to-marine transition									Comparison of trends by mean high salinity (psu)								
X	Y	Samples	n	Slope Equation	r	R^2^		*p*-value	X	Y	Samples	n	Slope Equation	r	R^2^		*p*-value
RK ≤ 15 to − 14.2	Sr/Ba-HAc	All	34	y = − 0.125 x + 2.14	0.72	0.52	M	< 0.001	Salinity	Sr/Ba-HAc	SG1–6	59	y = 0.105 x + 0.69	0.50	0.25	W	< 0.001
RK ≤ 15 to − 14.2	Sr/Ba-HAc	Shallow	18	y = − 0.161 x + 2.61	0.86	0.75	S	< 0.001	Salinity	Sr/Ba-HAc	SG1–5	36	y = 0.285 x − 0.31	0.85	0.73	M	< 0.001
RK ≤ 15 to − 14.2	Sr/Ba-NH_4_Ac	All	34	y = − 0.042 x + 1.0	0.67	0.45	W	< 0.001	Salinity	Sr/Ba-NH_4_Ac	SG1–6	59	y = 0.056 x + 0.69	0.32	0.10	N	0.01
RK ≤ 15 to − 14.2	Sr/Ba-NH_4_Ac	Shallow	18	y = − 0.047 x + 1.12	0.76	0.58	M	< 0.001	Salinity	Sr/Ba-NH_4_Ac	SG1–5	36	y = 0.245 x − 0.35	0.82	0.67	M	< 0.001
RK 110.6 to > 11	δ^13^C_org_	All	30	y = − 0.014 x − 25.37	0.53	0.28	W	0.003	Salinity	δ^13^C_org_	SG1–6	90	y = 0.069 x − 25.91	0.54	0.30	W	< 0.001
RK ≤ 15 to − 14.2	δ^13^C_org_	All	52	y = − 0.073 x − 24.42	0.41	0.17	N	0.002	Salinity	δ^13^C_org_	SG1–5	59	y = 0.159 x − 26.44	0.80	0.64	M	< 0.001
RK ≤ 15 to − 14.2	δ^13^C_org_	Shallow	33	y = − 0.074 x − 24.19	0.47	0.22	N	0.006									
RK ≤ 15 to − 14.2	dinocyst RA	All	33	y = − 0.397 x + 3.93	0.60	0.36	W	< 0.001	Salinity	Dinocyst RA	SG1–6	55	y = 0.187 x − 0.44	0.55	0.30	W	< 0.001
RK ≤ 15 to − 14.2	dinocyst RA	Shallow	26	y = − 0.246 x + 2.91	0.60	0.36	W	0.001									
RK ≤ 15 to − 14.2	dinocyst AA	All	33	y = − 60.35 x + 498.2	0.66	0.44	W	< 0.001	Salinity	Dinocyst AA	SG1–6	55	y = 23.48 x − 59.9	0.50	0.25	W	< 0.001
RK ≤ 15 to − 14.2	dinocyst AA	Shallow	26	y = − 35.56 x + 338.8	0.78	0.61	M	< 0.001									

In the sample column, “shallow” indicates that slope equations are derived using only surface and near-surface samples (taken from the upper 1 m of the sediment pile). Correlation coefficients (r), coefficients of determination (R^2^), and *p*-values are presented for all relations. Coefficients of determination are labelled as strong (R^2^ ≥ 0.75), moderate (0.5 ≤ R^2^ < 0.75), weak (0.25 ≤ R^2^ < 0.5), or negligible (0.1 ≤ R^2^ < 0.25) for the purpose of comparison. Only relations that are statistically significant (*p* < 0.05) are shown.

δ^13^C_org_ values increase seaward through the FMT (Fig. 2B). The relation between depositional position and δ^13^C_org_ is weak through the River (R^2^ = 0.28; Table 1) and negligible in the Delta (R^2^ = 0.17; Table 1). The relation between δ^13^C_org_ and depositional position in the Delta remains negligible if only surface and near surface samples are included (R^2^ = 0.22). C/N shows virtually no difference in values between the landward end of the FMT (River; 13.3 ± 1.8 (n = 6)) and the seaward end (Delta; 11.3 ± 2.4 (n = 26); Fig. 2B).

Dinocyst relative abundances show very low (< 1%) and very slightly increasing numbers of cysts seaward through the River (Fig. 2C) with relative abundances of 0.47% ± 0.48% (n = 22). Dinocyst relative abundances increase rapidly seaward through the Delta (Fig. 2D), although the correlation to river km is weak (R^2^ = 0.36) and does not improve if only surface and near surface samples are considered (R^2^ = 0.36; Table 1). Dinocyst absolute abundances also show very low and very slightly increasing numbers of cysts seaward through the River (Fig. 2C) with 81 ± 109 dinocysts g^−1^ (n = 22). Dinocyst absolute abundances increase rapidly seaward through the Delta (Fig. 2D), but correlate weakly to river km (R^2^ = 0.44). The correlation to river km improves markedly if only surface and near surface samples are considered (y = − 35.56x + 338.8, R^2^ = 0.61; Table 1).

### Salinity indicators

Sr/Ba-HAc, Sr/Ba-NH_4_Ac, and δ^13^C_org_ exhibit visually discernable trends from SG1 (0 psu) to SG5 (≤ 25 psu; Fig. 2 E–H), but these trends are less clear when samples from SG6 are included. Consequently, trends between salinity and the six sediment attributes are evaluated separately for SG1–5 and for SG1–6 (Table 1).

**Figure 3:**
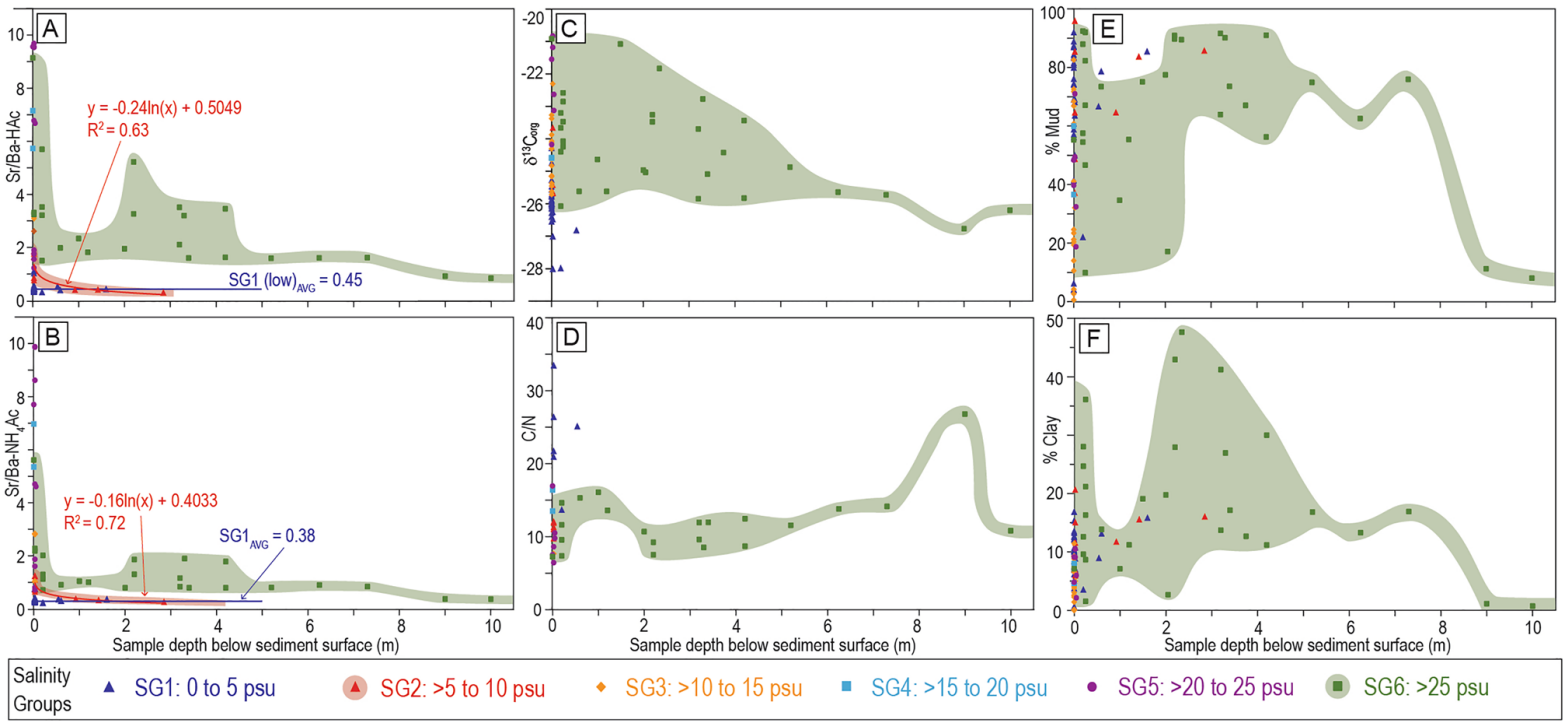
Graphs of changes in (**A–B**) Sr/Ba, (**C**) δ^13^C_org_, (**D**) C/N, (**E**) mud content, and (**F**) clay content with depth in the sediment and by Salinity Group. The coloured polygons are included to illustrate the distribution of data only.

The correlation between Sr/Ba-HAc and mean high salinity is weak (R^2^ = 0.25) for SG1–6 and increases markedly for SG1–5 (y = 0.285x − 0.31, R^2^ = 0.73; Fig. 2E). The correlation between mean high salinity and Sr/Ba-NH_4_Ac is negligible to no correlation (R^2^ = 0.1) for SG1–6 and moderate (y = 0.245x − 0.35, R^2^ = 0.67) for SG1–5. In SG6, both Sr/Ba ratios show a rapid decrease with depth in the sediment reaching apparent baseline values of 0.68 (Sr/Ba-NH_4_Ac) and 1.34 (Sr/Ba-HAc) by 4.5 m depth (Fig. 3A–B). Note that 4 of 5 samples below 4.5 m are from the same cored interval that was recovered from 134 m water depth (2011004PGC129, Fig. 1; Supplementary Data File A).

δ^13^C_org_ values correlate moderately well to salinity for SG1–5 (y = 0.16x − 26.44, R^2^ = 0.64; Fig. 2F), and weakly for SG1–6 (R^2^ = 0.3). In SG6, δ^13^C_org_ values decrease with depth in the sediment reaching an apparent baseline of approximately − 26 ‰ at 6 m (Fig. 3C); all samples below 6 m are derived from the same cored interval (2011004PGC129, Fig. 1). C/N shows no relation to salinity for SG1–6 and averages 11.5 ± 2.8. There is also no statistically significant relation between C/N and salinity for SG1–5 (Fig. 2F) and C/N shows no significant correlation to depth in the sediment (Fig. 3D).

Dinocyst relative abundance correlates weakly (R^2^ = 0.3) to salinity for SG1–6 and is constant at 0.67% ± 0.62% (n = 41) through SG1–5 (Fig. 2G). Of note, nearly all of the samples in SG1–5 (39 of 41) derive from the River segment of the FMT. Dinocyst absolute abundances show a weak (to negligible) correlation (R^2^ = 0.25) to salinity through SG1–6 and a more even distribution of 73 ± 99 dinocysts g^-1^ through SG1–5 (n = 41; Fig. 2H; Table 1).

### Attributes versus sediment characteristics

**Figure 4:**
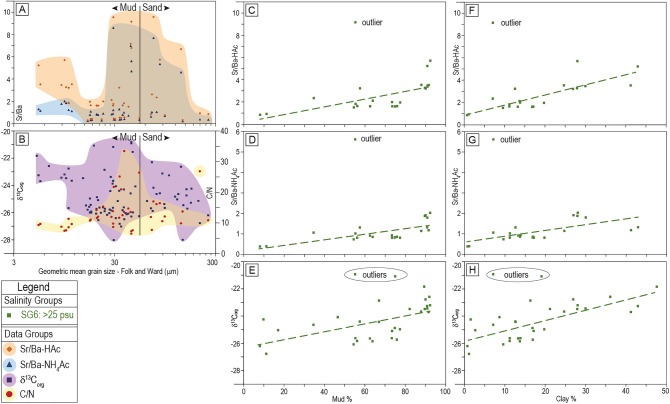
(**A**) Sr/Ba versus bulk grain size. (**B**) δ^13^C_org_ and C/N versus bulk grain size. Graphs of changes in (**C**) Sr/Ba-HAc, (**D**) Sr/Ba-NH_4_Ac, and (**E**) δ^13^C_org_ versus percent mud content for samples in Salinity Group 6 only. Graphs of changes in (**F**) Sr/Ba-HAc, (**G**) Sr/Ba-NH_4_Ac, and (**H**) δ^13^C_org_ versus percent clay content for samples in Salinity Group 6 only. Linear regression equations and R^2^ values are summarized in Table 2. The outlier in (**C–D**) and (**F–G**) is from the same sample taken from a Coastal area (MB21-S4, Supplementary Data File A), and the two outliers in (**E**) and (**H**) include the same Coastal sample and one derived from a cored interval. These outliers are not included in the slope equations and correlation coefficients presented in Table 2 and discussed in the text. The coloured polygons are included to illustrate the distribution of data only.

There is no obvious correlation between either Sr/Ba ratio and bulk grain size (Fig. 4A). Similarly, neither δ^13^C_org_ nor C/N correlates to bulk grain size (Fig. 4B). However, in the outer Delta region of the FMT (delta front and prodelta) and in many Coastal sites (SG6), four of six attributes show a significantly poorer correlation to salinity than in other salinity groups (Fig. 2) and assessing the cause of this requires correlation of attributes to both depth in the sediment (discussed previously; Fig. 3) and to sediment characteristics (Fig. 4; Table 2).

**Table 2:** Summary of statistics between the three sediment attributes (Sr/Ba-HAc, Sr/Ba-NH_4_Ac, and δ^13^C_org_) that show markedly different coefficients of determination when SG6 samples are excluded or included in linear regressions (Fig. 2, Table 1).

Comparison of trends by river km (RK)–Fluvial-to-Marine Transition									Comparison of trends by mean high salinity (psu)								
X	Y	Samples	n	Slope Equation	r	R^2^		*p*-value	X	Y	Samples	n	Slope Equation	r	R^2^		*p*-value
Mud%	Sr/Ba-HAc	All	21	y = 0.035 x + 0.16	0.68	0.46	W	< 0.001	Clay%	Sr/Ba-HAc	All	21	y = 0.092 x + 0.84	0.81	0.65	M	< 0.001
Mud%	Sr/Ba-NH_4_Ac	All	21	y = 0.013 x + 0.20	0.71	0.51	M	< 0.001	Clay%	Sr/Ba-NH_4_Ac	All	21	y = 0.028 x + 0.58	0.68	0.46	W	< 0.001
Mud%	δ^13^C_org_	All	29	y = 0.029 x − 26.34	0.61	0.37	W	< 0.001	Clay%	δ^13^C_org_	All	29	y = 0.076 x− 25.85	0.75	0.57	M	< 0.001

Samples from SG6 are compared to sediment characteristics, including percent mud and percent clay.

Sr/Ba-HAc correlates weakly to mud percent (R^2^ = 0.46; Fig. 4C), and this correlation increases markedly when Sr/Ba-HAc is compared to clay percent (y = 0.092x + 0.84, R^2^ = 0.65; Fig. 4F; Table 2). Sr/Ba-NH_4_Ac shows a moderate correlation to mud percent (y = 0.013x + 0.20, R^2^ = 0.51; Fig. 4D) and a weak correlation to clay percent (R^2^ = 0.46; Fig. 4G; Table 2). δ^13^C_org_ correlates weakly to mud percent (R^2^ = 0.37; Fig. 4E) and moderately to clay percent (y = 0.076x − 25.85, R^2^ = 0.57; Fig. 4H; Table 2).

## Discussion

The comparison of sediment attributes both along the FMT of the Fraser River Delta and as a function of salinity reveal several interesting trends. Trends along the FMT are differentiated from those related to salinity because sedimentation is significantly higher and salinity is significantly more variable along the FMT than in surrounding coastal areas.

### Sediment attributes as indicators of depositional position in the FMT

The comparison of sediment attributes to depositional position in the FMT reveals a significant increase in both Sr/Ba ratios and δ^13^C_org_ values at approximately River km 11 (Figs. 1 and 2). Dinocyst relative and absolute abundances also appear to increase from this point seaward. River km 11 correlates closely to the position of sustained brackish water in the Fraser River^28,37,38^, and so the response of the various sediment attributes seaward of RK11 appears to record the physical and/or chemical influence of *sustained* salinity on sedimentation. This hypothesis is supported by trends in the River (RK110.6 to 11), where 4 of 6 attributes (excluding δ^13^C_org_ and C/N) show little to no change in values regardless of depositional position. While this is expected where saltwater does not extend (landward of RK30), it is surprising in the region of saltwater incursion (RK30 to 11) and suggests that brackish water has a limited impact on sedimentation unless it is sustained. Further study is needed to confirm this.

Seaward of RK11, in the Delta region, Sr/Ba-HAc and Sr/Ba-NH_4_Ac both show good correlation to depositional position, particularly if only surface and near-surface samples are considered (Table 1). The response of Sr/Ba-HAc is three times greater than that of Sr/Ba-NH_4_Ac, suggesting that this is the preferred ratio for predicting depositional position in settings with sustained saltwater. The reason why Sr/Ba-HAc is larger than Sr/Ba-NH_4_Ac in saltwater environments is because both exchangeable and carbonate-bound Sr and Ba are extracted by acetic acid, while exchangeable Sr and exchangeable and barite-bound Ba are extracted by ammonium acetate. Because the Sr extracted by acetic acid is higher than that extracted by ammonium acetate, and the Ba extracted by ammonium acetate is higher than that extracted by acetic acid, the response of Sr/Ba-HAc exceeds that of Sr/Ba-NH_4_Ac in the same sediment.

Dinocyst relative and absolute abundances also show responses to depositional position through the Delta region of the FMT, although the correlation between these two variables is generally weak (Table 1). The exception to this are dinocyst absolute abundances when only surface and near-surface samples are considered; this relation is moderate. The moderate correlation of dinocyst absolute abundances to depositional position in surface and near surface samples is a direct comparison of sediment deposited under similar conditions and probably records the increased incorporation of dinocysts into marine sediment with time.

The general increase in δ^13^C_org_ through the FMT of the Fraser River records the transition from terrestrially-sourced organic matter (C_3_ plants and soil: − 26 to − 28‰) landward of RK 11 to increasingly marine-sourced organic matter (− 21 to − 23‰)^22^ seaward of RK11 (Fig. 2). Interestingly, the seaward increase in δ^13^C_org_ is not constant, and there appears to be a significant increase in δ^13^C_org_ at ~ RK60. The cause of this increase is not immediately apparent but could reflect an increase in C_4_ plant material (grasses) incorporated in sediment on the margins of the Fraser River and derived from the surrounding upper delta plain and farmland (Fig. 2). C/N exhibits no discernable trends, although this may reflect the paucity of data between ~ RK90 and RK10.

### Sediment attributes as indicators of salinity

The correlation of the six sediment attributes to mean high salinity shows more complicated trends than to depositional position (Fig. 2), and this reflects 1) the inclusion of samples derived from both the FMT and Coastal areas, and 2) the impacts of both depositional processes and sedimentological properties on attributes (Figs. 3 and 4). First, through SG 1–5, Sr/Ba-HAc, Sr/Ba-NH_4_Ac, and δ^13^C_org_ track changes in salinity reasonably well (Table 1) suggesting that all three attributes record physical and/or chemical influences of saltwater in sediment. Dinocyst relative and absolute abundances show no change from SG 1–5, but this probably reflects the paucity of data in SG3–5. Note that the two “outlier” values in SG4 and 5 (Fig. 2G–H) are from Coastal areas (Fig. 1B) and are near each other; they do not show any discernable relation to samples from the FMT and other Coastal samples.

The decrease in Sr/Ba with depth, and mainly in a single cored interval from 134 m water depth (Fig. 3A–B) is ascribed to the low clay content in the sediment below 4.5 m (Figs. 3F and 4F–G) and to possibly low carbonate content (lower Ca-HAc and inorganic carbon, Supplementary Data File A). The lower carbonate content in samples indicates reduced calcium carbonate shell material, which results in reduced adsorption of Sr and reduced isomorphic Sr extracted by HAc; this is manifested as reduced Sr/Ba^19^. The cause of reduced clay content and lower carbonate content with depth probably records variability in the amount of terrestrial organic material incorporated in rapidly buried fine-grained material^40,41^. Indeed, the Fraser Delta front and prodelta experience a wide range of gravity-driven flows that transport sediment from shallow water to deep^42–46^ and these flows commonly introduce terrestrial organic matter directly from the Fraser River or transport sediment from the tidal flats into the delta front and/or prodelta. In addition, periodic mass-wasting events transport terrestrial organic material offshore^47^. Terrestrial organic material may also be allocthonous and transported to the delta front from other coastal regions of western North America via deep-water renewal events^39,48^.

Interestingly, δ^13^C_org_ correlates reasonably well to clay percent (Table 2) suggesting a positive linkage. In the Fraser Delta, clay is deposited dominantly offshore and in deep water, while silt is the more common mud type deposited in the river, tidal flats, and delta front^49^. The deposition of clay in deep-water marine settings should also be where marine-sourced organic material is most prevalent, and the correlation between clay percent and ^13^C enrichment (Fig. 4H; Table 2) probably reflects this.

### Implications for paleosalinity

Of the various techniques compared herein, Sr/Ba-NH_4_Ac and especially Sr/Ba-HAc show the best response to increasing salinity and appear to correlate well when saline water is present and sustained in a depositional setting. However, our data also indicate that these ratios are impacted by a wide range of depositional processes, and low values should not be interpreted as indicating no or low-salinity conditions without considering alternate causes for their reduction. For example, neither ratio increases in the Fraser River where saline water incurs but is not sustained (RK30–11), and values are low in the delta front and prodelta when shallow-water sediment is transported into deeper water.

δ^13^C_org_ is highly variable through the FMT and does not appear to correlate to depositional position (Table 1); instead δ^13^C_org_ values reflect the dominance of terrestrial organic matter in deltaic settings. δ^13^C_org_ appears to be a reasonable indicator of salinity conditions, but again is impacted heavily by depositional processes, especially in deltas (Fig. 2F). The same is true for the relative and absolute abundances of dinocysts, which are also heavily impacted by river-derived sedimentation^10^. Consequently, our data suggest that it is not advisable to compare values from river-influenced settings (e.g., FMT, deltas) with those from non-river influenced settings (shorefaces, bays, and open marine). As well, interpreting salinity trends using sediment proxies is most effective if values are derived along a depositional profile and can be compared relatively.

## Methods

### Sample analyses

Grain size was measured for 98 samples using a Malvern Mastersizer 2000. Between 0.7 and 2 g of sediment was extracted from each sample and then treated with 30% H_2_O_2_ for 36 h to remove organic material. The supernatant was then pipetted off and the samples were mixed with 30 mL of 0.5% Sodium Hexametaphosphate solution and left for 24 h. Samples were then stirred using a magnetic mixer for 5 min and were placed in a sonic bath for 1 min. Following the sonic bath, samples were emptied into the Malvern Mastersizer and analyzed for grain size.

Sr/Ba was measured for 61 samples (+ 14 repeat analyses) including 38 from along the FMT. Sr/Ba was determined through selective extraction using both 10% acetic acid (Sr/Ba-HAc) and 1 M of ammonium acetate (Sr/Ba-NH_4_Ac) and following the methodology outlined in Wang, et al.^19,50^. First, the sample was dried and crushed till it passed through a 0.149 mm (100) mesh. Two, 0.1 g sub-samples were extracted from the crushed and dried sample and were placed in two 15 mL plastic centrifuge tubes. Ten mL of 10% acetic acid was added to one centrifuge tube and 10 mL of 1 M ammonium acetate to the other. The mixtures were stirred at room temperature (20–30 ℃) for 2 h, and then left to stand for 24 h (mixtures can also be centrifuged for 20 min at 4500 rpm to separate the solid and liquid). The supernatant was then used to analyze Sr and Ba using an ICP-AES. Alternatively, the supernatant can be diluted to one-tenth of its original concentration for analysis by ICP-MS.

δ^13^C_org_ values were determined for 90 samples, including 48 samples from Czarnecki, et al.^10^, and C/N (% total organic carbon / % total nitrogen) was determined for 42 of these samples. For the 42 new samples, samples were first cleaned used deionized water and then dried in an oven at 60 °C for 48 h. Clean and dried samples were pulverized and sieved through a 0.63 mm mesh, and 1 g of each sample was extracted and treated with 5 mL of 2 N hydrochloric acid (HCl) for approximately 16 h at room temperature to remove inorganic carbon^22^. A 40 mg sub-sample was extracted from each de-carbonated sample and was placed in a tin capsule in preparation for measuring total organic carbon and nitrogen contents. Elemental analyses were done using an elemental analyzer (vario MICRO cube elementar) in the Marine Geochemistry lab in the Institute of Oceanography, National Taiwan University, Taiwan. The standard used in elemental analysis is soil standard no. 502–062 Leco Reference Materials (%C = 0.924, %N = 0.093). All measurements were performed in duplicate and the relative error by multiple analyses of reference material was < 5%. The stable carbon isotope composition was measured using an elemental analyzer (Thermo Scientific Flash EA) connected with an isotope ratio mass spectrometer (Thermo Scientific Delta V Advantage). Carbon isotopic composition is presented as δ^13^C in the standard δ-notation in per mil (‰) with respect to Vienna Pee Dee Belemnite (VPDB). The measurements were calibrated with the standard reference material IAEA-CH-3 (δ^13^C = − 24.72 ± 0.05 ‰) and the analytical reproducibility for both δ^13^C is better than 0.2‰.

Palynomorph analyses include 45 from Czarnecki, et al.^10^ and 12 new samples (n = 57). The 12 new samples were processed at the Paleoenvironmental Laboratory, University of Minnesota, USA. Dinoflagellate cysts, pollen grains and spores were extracted using a standard dinocyst extraction method^51^. Approximately 3 cm^3^ of sediment were subsampled and oven-dried at ~ 40 °C and then weighed. Two calibrated tablets of *Lycopodium clavatum* spores (batch 140,119,321) were added to each sample to estimate palynomorph concentrations^52^. Samples were treated with room-temperature hydrochloric acid (10%) to dissolve carbonates, rinsed twice with reverse osmosis (RO) water, sieved through a 120 μm mesh and retained on a 15 μm nylon Nitex mesh to remove coarse and fine particles. Siliceous material was dissolved by using room-temperature hydrofluoric acid (48%) for up to two weeks. Samples were subsequently treated with hydrochloric acid (10%) to remove precipitated fluorosilicates, rinsed with RO water several times, gently sonicated for < 60 s, and sieved again through a 15 μm mesh sieve. After each step, samples were centrifuged at 3600 rpm for six minutes. Two drops of the residue were mounted between a slide and coverslip in glycerine jelly and marine palynomorphs were counted at 600 × magnification. Dinocyst and other palynomorphs were identified and counted using a Nikon Eclipse 80i transmitting light microscope. Cyst identification was made based on of published descriptions^53^. Palynomorphs are grouped into tree pollen, herbs and shrubs pollen, spores, and dinocysts. Dinocysts are subdivided into autotrophs and heterotrophs, and the relative abundances and concentrations of all dinocysts are calculated (Supplementary Data File A).

### Statistical methods

Sediment attribute data are divided into two discrete populations based on visual inspection of graphs. A break appears in nearly all datasets between values in the river (RK 110.6 to > 11; referred to herein as River) and those from the most seaward extend of the river, the tidal flats, delta front and prodelta (RK ≤ 11 to − 14.2; referred to herein as Delta). Means and standard deviations (and the slope equation for δ^13^C_org_) of samples along the River use all data between RK110.6 and > 11; however, slope equations for Delta samples are calculated from RK15 and seaward to ensure trends in the Delta population are continuous from the River population (i.e., the River population effectively acts as the y-intercept for the Delta population).

The correlation coefficient (r), coefficient of determination (R^2^), and *p*-value for each equation is calculated for the River and Delta populations and for all attributes using the Analysis ToolPak in Microsoft® Excel and using linear regression only. Coefficients of determination are labelled as strong (R^2^ ≥ 0.75), moderate (0.5 ≤ R^2^ < 0.75), weak (0.25 ≤ R^2^ < 0.5), or negligible (0.1 ≤ R^2^ < 0.25) for the purpose of comparison. For linear regression equations with an R^2^ < 0.1 (i.e., slope equation explains < 10% of the data), values are reported as mean ± standard deviation only. Statistically insignificant slope equations (*p* ≥ 0.05), equations where there is a ≥ 5% chance that there is no relationship between the two variables, are not reported herein.

Outliers are identified in all datasets and are excluded from quantitative assessments (linear regression and mean ± standard deviation; Tables 1 and 2). This is done to resolve underlying trends in relatively low-n datasets, and to avoid skewing trends considerably based on one or two datapoints. Note that the slope equations listed in Table 1 are only valid when compared together (i.e., relatively) because 1) river km data is unique to the Fraser River and 2) mean high salinity is representative because of significant salinity changes associated with tidal fluctuations, precipitation, and river discharge.

## Supplementary Information

Supplementary Information.

## Data Availability

All data generated or analysed during this study are included in this published article [and its supplementary information files].
